# Correlation of systemic immune-inflammation index and functional status of living score with disease activity in patients with rheumatoid arthritis

**DOI:** 10.1016/j.clinsp.2025.100815

**Published:** 2025-11-11

**Authors:** Chuan Yang, Yu Shu, Jie Luo, DongSheng Wang

**Affiliations:** a School of Clinical Medicine, Southwest Medical University, Luzhou City, Sichuan Province, China; b Department of Clinical Laboratory, Chengdu Wenjiang District People's Hospital, Chengdu City, Sichuan Province, China

**Keywords:** Systemic immune-inflammation index, Dysfunction, Rheumatoid arthritis, Disease activity, Immune disorders

## Abstract

**Objective:**

This study aims to investigate the correlation between Systemic Immune-Inflammation (SII) index and Health Assessment Questionnaire (HAQ) scores and disease activity in patients with Rheumatoid Arthritis (RA).

**Methods:**

Clinical and laboratory data of 132 RA patients, including SII index, disease activity scores, and HAQ scores were collected. The patients were categorized as moderate disease activity and High Disease Activity (HAD). Univariate and multivariate linear analyses were performed to find correlations between the SII index and HAQ with DAS28-CRP and DAS-ESR.

**Results:**

The HAD-RA cohort had a higher SII index (1.28 [IQR: 0.97, 2.10] vs. 0.89 [IQR: 0.66, 1.27], *p* < 0.001) and HAQ (1.80 [1.40, 2.10] vs. 1.05 [IQR: 0.71, 1.40], *p* < 0.001). A relationship was found between the SII index, HAQ scores, and disease activity, where individuals with worse functional status had higher disease activity. Multifactor linear analysis further validated the independent correlation between the SII index and HAQ scores with RA disease activity. Decreased hemoglobin was a relevant factor affecting DAS28-CRP and DAS-ESR. The AUC of the SII index was 73.69 with a cutoff value of 0.93, and the AUC of HAQ was 84.41 with a cutoff value of 1.45. The combination of the two resulted in an AUC of 85.76, which increased the sensitivity and specificity to 83.99 % and 94.00 %, respectively.

**Conclusion:**

Both the SII index and the HAQ scores are significantly associated with disease activity in RA patients. These insights support the accurate assessment of the disease, the design of personalized treatment plans, and the evaluation of treatment efficacy.

## Highlights

• SII index and HAQ scores are significantly associated with RA disease activity.
• SII and HAQ combination increases AUC to 85.76 for disease activity prediction.
• Decreased hemoglobin was a relevant factor affecting DAS28-CRP and DAS-ESR.

## Introduction

Rheumatoid Arthritis (RA) is a long-term autoimmune disorder that mainly affects the joints, leading to persistent synovitis that triggers a series of processes damaging cartilage and bone. This pathological process frequently results in joint deformity and impaired function.^1^ Epidemiological data show that RA affects between 0.3 % and 1 % of the global population, with a higher incidence in women and a common onset age range of 40 to 60.^2^ Although the detailed causes of RA are not entirely elucidated, research indicates that a complex interaction of genetic factors, environmental triggers, and immune system dysfunction is involved in its pathogenesis.^3^ Beyond joint health, RA can also affect various systems in the body, such as the cardiovascular, respiratory, digestive, and neurological systems, leading to pathophysiological changes that can affect patients' quality of life and life expectancy.^4^ Disease activity greatly influences the prognosis of RA patients, highlighting the importance of identifying markers to assess this activity for early prognosis and medical decisions.

Indicators that are commonly used in clinical practice to assess RA disease activity include ACR20/50/70,^5^ the remission criteria for RA recommended by the American College of Rheumatology/European League against Rheumatism (ACR/EULAR).^6^ Disease Activity Score (DAS).^7^ Simplified Disease Activity Index (SDAI), and Clinical Disease Activity Index (CDAI).^8^ RA treatment targets low disease activity or remission by employing early and supportive strategies. These indices are suggested in clinical environments to evaluate treatment success and adjust therapeutic plans.^9^^,^^10^ SDAI and CDAI are especially beneficial for outpatient monitoring due to their simplicity and rapid results.^11^ The DAS28 score, which integrates clinical, laboratory, and patient-reported data, is often used in clinical settings to assess disease activity.^12^

In RA, there is a complex interaction among immune cells and molecules, especially T-cells, B-cells, and cytokines.^13^ The Systemic Immune-Inflammation (SII) index, calculated using neutrophil, lymphocyte, and platelet counts (SII=[PLT×NEU]/LYM), is suggested as a comprehensive marker that could provide a broader view of systemic inflammation and immune status. The SII index is higher in patients with active RA, making it a trustworthy indicator for distinguishing active RA.^14^

RA leads to joint pain, swelling, and deformities, limiting joint mobility and muscle strength, which in turn diminishes the ability to perform daily activities.^15^ The Health Assessment Questionnaire (HAQ) can quantify the impact of the disease on daily activities, social functioning, and mental health. The ability of HAQ scores to independently predict long-term remission in RA patients is well-established.^16^ When combined with disease activity, these scores provide a more detailed picture of the health status and needs of RA patients.

This study was to investigate in depth the correlation between the SII index and HAQ scores and disease activity in RA patients. Integrating the SII index with existing indices and HAQ scores in the evaluation system enables early identification of changes in disease activity, timely adjustments to treatment plans, slowing disease progression, and enhancing patients' quality of life.

## Materials and methods

### Subjects

This study included 132 patients at Chengdu Wenjiang District People's Hospital from January 2024 to January 2025. These patients were diagnosed with RA according to the 2010 ACR/EULAR classification criteria. Adult patients with complete clinical information were included. Those with the following characteristics were excluded: 1) Comorbid severe cardiovascular disease, renal insufficiency, and chronic obstructive pulmonary disease; 2) Malignant tumors; and 3) Pregnancy or lactation. The study was approved by the Ethics Committee of Chengdu Wenjiang District People's Hospital (n° LW-KY-2025–019), and all patients or family members signed an informed consent form. This study followed the STROBE guidelines.

### Data collection

In this single-center study, a cross-sectional retrospective approach was used to collect demographic and clinical information from the medical records of patients who met the criteria. General information included gender, age, disease duration, diagnosis, and previous treatment. Fasting venous blood was collected. Routine indices were measured, including Hemoglobin (Hb), NEU, LYM, PLT, red blood cell distribution width, and Mean Platelet Volume (MPV). Neutrophil-to-Lymphocyte Ratio (NLR), Platelet-to-Lymphocyte Ratio (PLR), and SII index were calculated. SIIindex=[(PLT×NE/LYM)/1000]. Moreover, blood Erythrocyte Sedimentation Rate (ESR) and C-Reactive Protein (CRP) were recorded. Three subtypes of Rheumatoid Factor (RF-IgA, RF-IgG, and RF-IgM) and anti-cyclic citrullinated protein were also determined. Joint involvement was assessed according to Tender Joint Count (TJC) and Swollen Joint Count (SJC) in 28 joints (including hands, MCP1–5, PIP1–5, wrists, elbows, shoulders, and knees).

### DAS28-CRP and Das28-ESR

RA disease activity was measured using the DAS28-CRP. The DAS28-ESR score was calculated based on TJC, SJC, ESR, and the patient's general health (usually derived from the visual analog scale). DAS28−ESR=0.56×(TJC)−2+0.28×(SJC)−2+0.7×ln(ESR)+0.014×(PGA). DAS28−CRP=0.56×(TJC)−2+0.28×(SJC)−2+0.36×ln(CRP+1)+0.014×(PGA)+0.96. A DAS28 score of 2.6 to less than 3.2 suggests Low Disease Activity (LDA), 3.2 to 5.1 indicates Moderate Disease Activity (MDA), and above 5.1 indicates High Disease Activity (HDA).

### HAQ scores

The HAQ scale consists of 20 questions related to activities of daily living, divided into eight subcategories: dressing, grooming, eating, arising, opening things, walking, reaching objects, maintaining hygiene, maintaining grip, and performing daily activities. Each item is scored on a 3-point scale: 0 for no difficulty, 1 for some difficulty, 2 for major difficulty, and 3 for inability to complete.

### Statistics and analysis

Statistical analyses were performed using SPSS 26.0 (IBM, New York, USA) and *R* software package 4.0.5. Data normality was assessed via the Shapiro-Wilk test. For normally distributed data, results are presented as mean ± standard deviation, and Student’s *t*-test was used for group comparisons. For skewed distributions, continuous variables are reported as median (Interquartile Range [IQR]), and group comparisons were conducted using the Mann-Whitney *U* test. Categorical data are expressed as frequency (n) and proportion (%), with group differences analyzed via the chi-square test. Using Spearman’s rank correlation analysis, the relationships among SII index, HAQ scores, and DAS28 in RA patients were evaluated, with p-values adjusted using the Bonferroni correction. In the *R* language, univariate and multivariate linear regression analyses were conducted to determine factors linked to DAS28. The ROC curve and AUC assessed the discriminative ability of the SII index and HAQ on MDA-RA and HAD-RA groups. The maximum Youden index was calculated to obtain the cutoff value, as well as the sensitivity and specificity of the cutoff value. Statistical significance was set at *p* < 0.05.

## Results

### Clinical characteristics of patients with RA

From January 2024 to January 2025, a total of 324 patients were diagnosed with RA in Chengdu Wenjiang District People's Hospital, including 226 patients with complete clinical and laboratory data, 190 RA patients with complete DAS data, 149 patients with HAQ assessment, 4 patients with other rheumatic diseases, 10 patients with severe cardiovascular disease, chronic obstructive pulmonary disease, renal insufficiency, and other organic diseases, 2 cases with malignant tumor, and 1 case during pregnancy or lactation. Eventually, 132 patients were included in the analysis, including 50 MDA-RA and 82 HAD-RA, with baseline information as shown in Table 1. The median age for the HAD-RA cohort was 59.0 years (IQR: 52.1, 65.6), surpassing the median age of the MDA-RA cohort, which was 53.3 years (IQR: 49.6, 62.3) (*p* = 0.048). Hb was lower in the HAD-RA cohort than in the MDA-RA cohort (*p* = 0.001). NEU, PLT, NLR, and PLR were all significantly higher in the HAD-RA cohort than in the MDA-RA cohort (all *p* < 0.05). In the HAD-RA cohort, there were higher SJCs and TJCs, increased PGA, elevated ESR and CRP levels, and higher DAS28 scores (all *p* < 0.001).

**Table 1:** Baseline characteristics of patients with MDA-RA and HDA-RA.

Data	MDA-RA (*n* = 50)	HAD-RA (*n* = 82)	p-value
Gender (F/M)	30/20 (60.0 %/40.0 %)	55/27 (67.07 %/32.93 %)	0.41
Age, y	53.3 (49.6, 62.3)	59.0 (52.1, 65.6)	0.048
Disease duration, Months	60 (13, 159)	75 (14, 136)	0.694
Hb, g/L	106.3 ± 17.0	96.3 ± 18.6	0.001
WBC, × 109/L	6.3 (5.3, 7.7)	6.5 (5.3, 8.2)	0.842
NEU, × 109/L	4.4 (3.4, 5.7)	5.0 (3.7, 6.6)	0.018
LYM, × 109/L	1.4 (1.2, 2.0)	1.3 (0.9, 1.8)	0.073
PLT, × 109/L	290.5 (246.5, 383.5)	363.5 (276.5, 435.8)	0.002
RDW, %	14.6 ± 2.3	14.7 ± 2.0	0.819
MPV, fL	10.3 ± 1.5	10.1 ± 1.1	0.156
NLR	2.9 (2.3, 3.6)	3.7 (2.6, 5.5)	<0.001
PLR	197.5 (153.1, 288.9)	278.6 (177.9, 426.1)	0.006
SJC, n	2.0 (1.0, 4.5)	10.0 (5.0, 21.0)	<0.001
TJC, n	2.5 (2.0, 5.5)	14.0 (8.0, 24.00)	<0.001
PGA (0‒100 mm)	50.0 (32.5, 58.5)	68.5 (50.0,75.5)	<0.001
ESR, mm/h	61.0 (39.0, 88.5)	84.0 (59.0, 100.0)	<0.001
CRP, mg/dL	29.0 (11.2, 73.6)	56.6 (27.6, 102.3)	0.005
DAS28-CRP	4.3 ± 0.6	6.7 ± 1.1	<0.001
DAS28-ESR	4.9 ± 0.8	7.0 ± 1.1	<0.001
RF-IgG+, n ( %)	32 (64.0 %)	52 (63.41 %)	0.946
RF-IgA+, n ( %)	26 (52.0 %)	43 (52.44 %)	0.961
RF-IgM+, n ( %)	34 (68.0 %)	60 (65.22 %)	0.738
anti-CCP+, n ( %)	40 (80.0 %)	72 (87.8 %)	0.225

Hb, Hemoglobin; NEU, Neutrophil; LYM, Lymphocyte; PLT, Platelet Count; RDW, Red Blood Cell volume distribution Width; MPV, Mean Platelet Volume; NLR, NEU/LYM Ratio; PLR, PLT/LYM Ratio; TJC, Tender Joint count; SJC, Swollen Joint Count; ESR, Erythrocyte Sedimentation Rate; CRP, C-Reactive Protein. Count data are expressed as n ( %), and measures are expressed as x ± s or M (IQR).

### SII index and HAQ scores in patients with RA

**Fig. 1:**
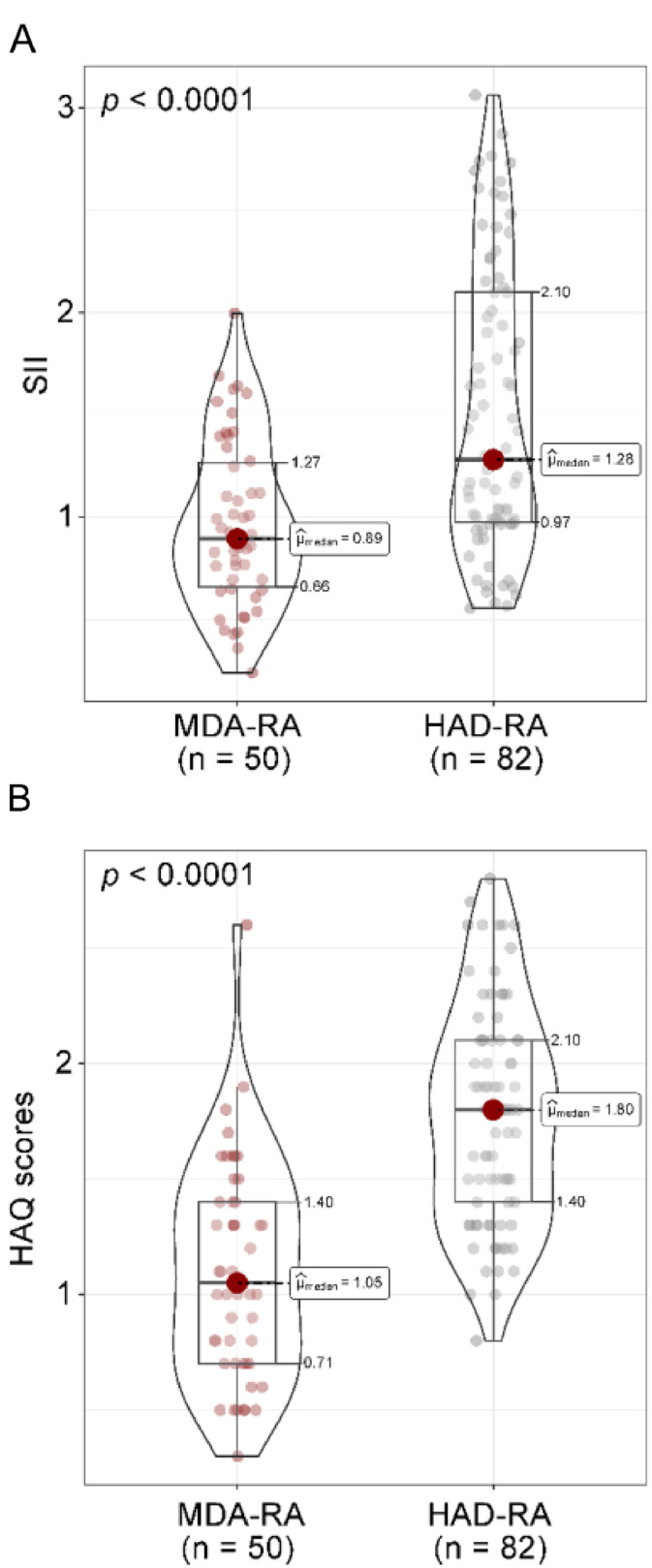
Comparison of SII index and HAQ scores in patients with RA. (A) SII index; (B) HAQ scores. *p* < 0.05 was statistically significant. MDA-RA refers to the Rheumatoid Arthritis (RA) group with moderate disease activity (*n* = 50), while HAD-RA denotes the RA group with relatively high disease activity (*n* = 82). The Mann-Whitney *U* test was employed to analyze the differences in SII index and HAQ scores between the two groups. The median (μ) is presented, and the results are shown as p-values corrected by Bonferroni.

First, the SII index was computed for all patients. The median SII index was 0.89 (IQR: 0.66–1.27) in the MDA-RA group compared to 1.28 (IQR: 0.97–2.10) in the HDA-RA group (*p* < 0.001). According to the HAQ scores, the HDA-RA group had a worse functional status, with a median score of 1.80 (IQR: 1.40‒2.10), while the MDA-RA group had a median score of 1.05 (IQR: 0.71‒1.40) (Fig. 1A‒B). These results demonstrate that elevated SII index and HAQ scores are associated with higher disease activity in RA.

### Linear analysis of correlates affecting disease activity in RA

**Fig. 2:**
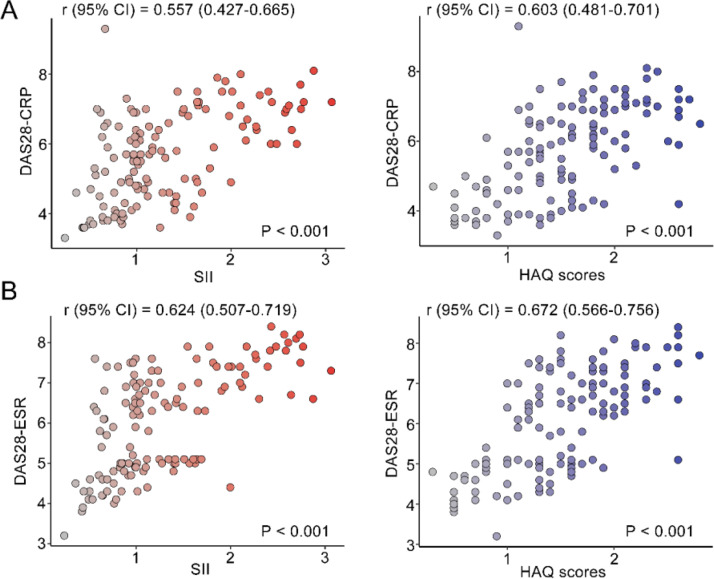
Correlation analysis of disease activity level with SII index and daily functional status. (A) Correlation of DAS28-CRP with SII index and HAQ scores; (B) Correlation of DAS28-ESR with SII index and HAQ scores. r indicates the correlation coefficient. Coefficients were categorized as weak for values between 0.3 and 0.5, moderate for 0.5 to 0.7, and strong for 0.7 to 1.0; *p* < 0.05 was statistically significant.

**Fig. 3:**
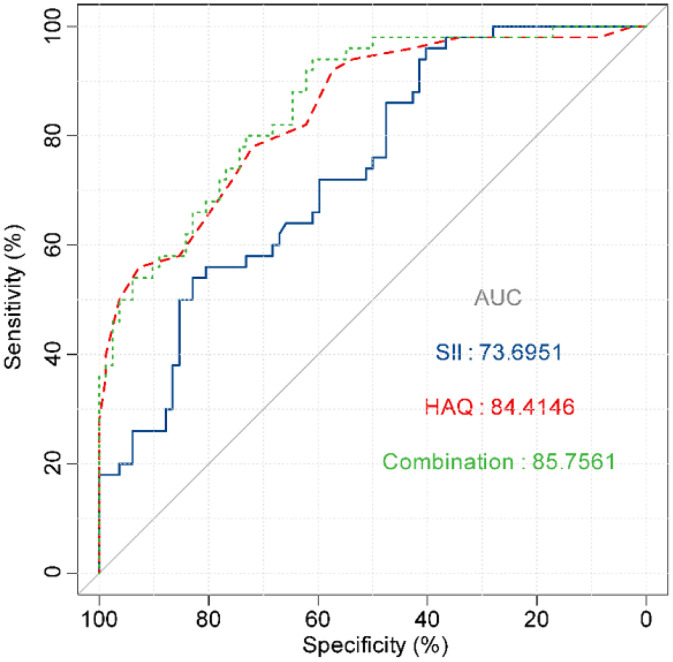
Discriminatory efficacy of the SII index and HAQ in evaluating disease activity in RA patients. AUC denotes the area under the curve and *p* < 0.001 for all analyses.

The correlation between DAS28 score and SII index, and HAQ scores was analyzed by univariate analysis. DAS28 score calculated based on CRP had a moderate positive correlation with the SII index (*r*95 % CI = 0.557 [0.427‒0.665], *p* < 0.001) and HAQ (*r*95 % CI = 0.603 [0.481‒0.701], *p* < 0.001) (Fig. 2A). DAS28 calculated based on ESR scores also had moderate positive correlations with the SII index (*r*95 % CI = 0.624 [0.507‒0.719], *p* < 0.001), and HAQ (*r*95 % CI = 0.672 [0.566‒0.756], *p* < 0.001) (Fig. 2B). Elevated age and lower Hb were linearly correlated with higher DAS28-CRP by univariate linear analysis; The multivariate linear analysis indicated that Hb had a negative correlation with DAS28-CRP and a positive correlation with both the SII index and HAQ scores (Table 2). Univariate and multivariate linear analyses also showed similar results for DAS28-ESR (Table 3). ROC curves and AUC area were used to evaluate the efficacy of the SII index and HAQ scores in differentiating the MDA-RA and HAD-RA cohorts (Fig. 3). The results showed that both the SII index and HAQ scores were effective in identifying patients with different levels of disease activity. The AUC of the SII index was 73.69, with a sensitivity of 82.93 % and a specificity of 54.00 % at a cut-off value of > 0.93; and the AUC of HAQ was 84.41, with a sensitivity of 71.95 % and a specificity of 78.00 % at a cut-off value of > 1.45. Although the combination of the two did not significantly increase the AUC (85.76), the sensitivity and specificity increased to 83.99 % and 94.00 %, respectively.

**Table 2:** Univariate and multivariate linear regression analysis of factors associated with disease activity score DAS28-CRP in RA patients.

Variables	Univariate					Multivariate				
	β	S.E	*t*	p	β (95 % CI)	β	S.E	*t*	p	β (95 % CI)
Age	0.03	0.01	2.45	**0.016**	0.03 (0.01 ∼ 0.06)	0	0.01	0.1	0.917	0.00 (−0.02 ∼ 0.02)
Hb	−0.03	0.01	−4.48	<0.001	−0.03 (−0.04 ∼ −0.01)	−0.01	0	−2.51	**0.013**	−0.01 (−0.02 ∼ −0.01)
SII	1.11	0.14	7.65	<0.001	1.11 (0.82 ∼ 1.39)	0.57	0.18	3.19	**0.002**	0.57 (0.22 ∼ 0.93)
HAQ	1.38	0.16	8.61	<0.001	1.38 (1.06 ∼ 1.69)	0.86	0.19	4.47	<0.001	0.86 (0.48 ∼ 1.24)

CI, Confidence Interval; Hb, Hemoglobin; SII, Systemic Immune-Inflammation Index; HAQ, Health Assessment Questionnaire.

**Table 3:** Univariate and multivariate linear regression analysis of factors associated with disease activity score DAS28-ESR in AR patients.

Variables	Univariate					Multivariate				
	β	S.E	*t*	p	β (95 % CI)	β	S.E	*t*	p	β (95 % CI)
Age	0.03	0.01	2.46	**0.015**	0.03 (0.01 ∼ 0.05)	0	0.01	−0.17	0.864	−0.00 (−0.02 ∼ 0.02)
Hb	−0.02	0.01	−4.1	**<0.001**	−0.02 (−0.04 ∼ −0.01)	−0.01	0	−2.07	**0.04**	−0.01 (−0.02 ∼ −0.01)
SII	1.22	0.13	9.07	**<0.001**	1.22 (0.95 ∼ 1.48)	0.66	0.16	4.09	**<0.001**	0.66 (0.35 ∼ 0.98)
HAQ	1.53	0.15	10.31	**<0.001**	1.53 (1.24 ∼ 1.82)	0.99	0.18	5.65	**<0.001**	0.99 (0.65 ∼ 1.33)

CI, Confidence Interval; Hb, Hemoglobin; SII, Systemic Immune-Inflammation Index; HAQ, Health Assessment Questionnaire.

## Discussion

The study ultimately enrolled 132 participants, consisting of 50 with MDA-RA and 82 with HAD-RA. Those diagnosed with HAD-RA demonstrated a higher SII index and a decline in daily living functional status. Multivariate linear analysis showed significant linear correlations between the SII index and HAQ scores with DAS28-CRP and DAS28-ESR.

Effectively managing RA depends on accurately assessing disease activity. This assessment is essential for informing treatment strategies, predicting long-term outcomes, and is directly connected to the patient's quality of life, extending beyond simply measuring symptom severity. By evaluating joint swelling, pain, stiffness, fatigue, and lab and imaging outcomes, disease activity offers a comprehensive reflection of RA patients' disease status,^17^ and facilitates treatment decisions. DAS28-CRP and DAS28-ESR are effective instruments for measuring disease activity in RA patients, helping to evaluate their condition and customize treatment plans. Hospitalized RA patients in this study experienced MDA and HDA, with LDA not being detected. Reflecting the established RA epidemiology, the present study’s cohort demonstrated a female majority, with a ratio of approximately 3:1.^18^ This difference is often credited to the immunomodulatory effects of sex hormones, like estrogen. This gender difference may be related to the immunomodulatory role of sex hormones. Estrogen and progesterone might contribute to the development of RA, particularly during menopause and after childbirth, when the risk and severity of RA rise.^19^ Both the age of RA onset and the patient's biological age could influence disease activity and clinical outcomes. Specifically, RA patients who develop the condition at an older age exhibit higher DAS28 scores, potentially linked to peripheral blood CD4^+^ *T*-cell activation.^20^ The present study also showed that the age in the HAD cohort was slightly greater than in the MAD cohort (*p* = 0.048).

RA is driven by immune system dysregulation, leading to ongoing inflammation that eventually causes joint damage.^21^, ^22^, ^23^ Although the SII index (based on neutrophil, lymphocyte, and platelet calculations) significantly correlates with disease activity, it is also important to recognize the nonspecific nature of its constituent parameters: these blood cell parameters can be affected by infection, stress, or other inflammatory states and are not RA-specific markers.^24^ However, the SII index may provide unique clinical value in that it simultaneously reflects a state of immune imbalance with neutrophil activation, lymphocyte depletion, and platelet involvement.^25^ The SII index is significantly elevated in patients with RA and is strongly associated with disease activity.^14^ The prognostic nutritional index (an index calculated on the basis of serum albumin and peripheral lymphocytes) proves to be a valid predictor of disease activity in patients with RA, and it correlates with inflammatory markers such as CRP and ESR.^26^ The present study further confirmed that the SII index was moderately positively correlated with both DAS28 scores (DAS28-CRP: *r* = 0.557, 95 % CI 0.427‒0.665; DAS28-ESR: *r* = 0.624, 95 % CI 0.507‒0.719) in patients with MDA/HDA-RA. This reinforces the potential of the SII index as a valid indicator of systemic inflammatory load in RA patients, especially its stronger correlation with ESR-based DAS28, which may be related to the fact that ESR itself is influenced by a variety of blood cell factors.

In addition to inflammatory markers, patient-reported functional status is a central goal in the management of RA. The HAQ is one of the gold standards for assessing functional limitations in daily life.^27^ The results of this study are consistent with previous studies showing that high disease activity was significantly associated with worse HAQ scores. More importantly, HAQ shared a moderate-strong positive correlation with DAS28 scores (DAS28-CRP: *r* = 0.603, 95 % CI 0.481‒0.701; DAS28-ESR: *r* = 0.672, 95 % CI 0.566‒0.756), and its correlation with DAS28-ESR was stronger than that of SII index (*r* = 0.672 vs. *r* = 0.624). This reaffirms the irreplaceable value of the HAQ in capturing the actual impact of disease on patient function. When age was factored into the linear analysis, the SII index and HAQ scores remained important determinants of DAS28-CRP and DAS28-ESR. Notably, reduced Hb was also a factor affecting disease activity in RA patients. It has been shown that nutritional status is also an influencing factor for the functional status of RA patients. Patients who are malnourished often show greater functional impairment and increased disease activity, highlighting the significance of nutritional management in the treatment of RA patients.^28^ Based on these findings, an integrated monitoring framework was proposed: in clinical practice, simultaneous monitoring of inflammatory markers (e.g., SII index, CRP, ESR) and functional status (HAQ) may provide a more comprehensive assessment of the disease, with elevated SII index suggesting the potential need for intensive anti-inflammatory therapy and elevated HAQ scores strongly suggesting that the regimen may need to be adjusted to improve functioning and quality of life even when inflammatory markers have been controlled. When both improve, maintenance or adjustment (e.g., dose reduction) of the treatment regimen may be considered. This two-dimensional assessment strategy helps to achieve more individualized RA management goals.

This study has some limitations. With a sample size of 132 cases from a single center, the study's findings might not entirely capture the general characteristics of RA patients. RA exhibits a high degree of heterogeneity, resulting in significant differences in clinical presentations, disease course, and treatment responses across different individuals. This may lead to differences in the correlation of the SII index and HAQ scores with disease activity among different patients, thus affecting the accuracy and reliability of the study results. RA is a chronic disease, and its disease activity may change over time. The correlation between the SII index and HAQ scores and disease activity may also be affected by disease progression, response to treatment, and other factors. Therefore, a single cross-sectional study may not be able to fully reflect the dynamic relationship between these indices. Future multicenter prospective cohort studies with longitudinal data are needed to validate these findings. In summary, limitations such as the diversity and complexity of assessment metrics, disease heterogeneity, sample selection and risk of bias, timeliness and dynamics of studies, and challenges of interdisciplinary collaboration and data integration need to be fully considered. In future studies, a multicenter, large-sample-size prospective study tracking the immune and nutritional dynamics of patients before and after treatment is necessary for a more complete understanding of the correlation between disease activity and immunity and nutrition in RA patients.

## Conclusion

RA patients show a significant association between the SII index and HAQ scores and disease activity. The SII index can more objectively reflect the systemic inflammatory load in RA patients and is strongly correlated with disease activity. Alternatively, the HAQ scores evaluate the degree to which daily activities and quality of life are impaired, showing a strong correlation with disease activity. It is important to emphasize that the SII index assesses the biological drivers of inflammation, whereas the HAQ assesses the actual impact of the disease on the patient's life (functional outcome). They are complementary rather than substitutes. These findings provide new perspectives and rationale for the clinical management of RA, and help physicians to more accurately assess patients' conditions, formulate individualized treatment plans, and monitor treatment effects. In the future, the value of the SII index and HAQ scores in the prognostic assessment of RA, as well as their integrated use with other disease activity assessment methods, can be further explored.

## Funding

Not applicable.

## Authors’ contributions

Chuan Yang designed the research study. Chuan Yang and Yu Shu performed the research. Jie Luo and DongSheng Wang provided help and advice. Yu Shu and Jie Luo analyzed the data. Chuan Yang wrote the manuscript. DongSheng Wang reviewed and edited the manuscript. All authors contributed to editorial changes in the manuscript. All authors read and approved the final manuscript.

## Data availability

The datasets used and/or analyzed during the present study are available from the corresponding author on reasonable request.

## Ethics approval

The present study was approved by the Ethics Committee of Chengdu Wenjiang District People's Hospital (n° LW-KY-2025–019), and written informed consent was provided by all patients prior to the study start. All procedures were performed in accordance with the ethical standards of the Institutional Review Board and the Declaration of Helsinki, and its later amendments or comparable ethical standards.

## Conflicts of interest

The authors declare no conflicts of interest.
